# Merkel Cell Carcinoma: An Updated Review Focused on Bone and Bone Marrow Metastases

**DOI:** 10.3390/cancers17132253

**Published:** 2025-07-06

**Authors:** Biagio Scotti, Elisabetta Broseghini, Costantino Ricci, Barbara Corti, Costanza Viola, Cosimo Misciali, Carlotta Baraldi, Sabina Vaccari, Martina Lambertini, Federico Venturi, Elisabetta Magnaterra, Aurora Alessandrini, Tiziano Ferrari, Massimo Lepri, Gabriele Argenziano, Barbara Melotti, Elena Campione, Davide Campana, Manuela Ferracin, Emi Dika

**Affiliations:** 1 Department of Medical and Surgical Sciences, Alma Mater Studiorum, University of Bologna, 40126 Bologna, Italy; federico.venturi12@unibo.it (F.V.); elisabetta.magnaterra@unibo.it (E.M.); tiziano.ferrari3@studio.unibo.it (T.F.); massimo.lepri@studio.unibo.it (M.L.); gabriele.argenziano@unibo.it (G.A.); davide.campana@unibo.it (D.C.); manuela.ferracin@unibo.it (M.F.); emi.dika3@unibo.it (E.D.); 2 Dermatology Unit, IRCCS Azienda Ospedaliero-Universitaria di Bologna, 40138 Bologna, Italy; cosimo.misciali@aosp.bo.it (C.M.); carlotta.baraldi@aosp.bo.it (C.B.); sabina.vaccari@aosp.bo.it (S.V.); mlambertini@hotmail.it (M.L.); aurora.alessandrini.30@gmail.com (A.A.); 3 IRCCS Azienda Ospedaliero-Universitaria di Bologna, 40138 Bologna, Italy; elisabett.broseghin2@unibo.it; 4 Pathology Unit, DIAP-Dipartimento InterAziendale di Anatomia Patologica di Bologna, Maggiore Hospital AUSL Bologna, 40133 Bologna, Italy; costanricci@gmail.com; 5 Pathology Unit, IRCCS Azienda Ospedaliero-Universitaria di Bologna, 40138 Bologna, Italy; babara.corti@aosp.bo.it; 6 Division of Cardiovascular and Interventional Radiology, Medical University of Vienna, 1090 Vienna, Austria; costanza.viola@gmail.com; 7 Oncology Unit, IRCCS Azienda Ospedaliero-Universitaria di Bologna, 40138 Bologna, Italy; barbara.melotti3@unibo.it; 8 Dermatology Unit, Department of Systems Medicine, University of Rome Tor Vergata, 00133 Rome, Italy; elena.campione@uniroma2.it; 9 Department of Medical Oncology, IRCCS Azienda Ospedaliero-Universitaria di Bologna, 40138 Bologna, Italy

**Keywords:** Merkel cell carcinoma, metastasis, bone, bone marrow, diagnosis, dermoscopy, reflectance confocal microscopy, treatment, therapy, radiotherapy, etiology, origin, MCC, MCCUP, RCM

## Abstract

Background/objectives: Despite advancements in early diagnosis and clinical practices guided by standardized care protocols, Merkel cell carcinoma (MCC) is marked by an unfavorable prognosis with a 5-year relative survival rate of 65%, based primarily on data collected prior to the introduction of immunotherapy. Regional nodal metastases affect 40–50% of MCC patients, while approximately 33% experience distant dissemination. Among these, bone and bone marrow metastases are particularly notable, although the characteristics and clinical implications of this metastatic disease in MCC remain poorly understood. Methods: A comprehensive review was conducted using the Medline database (via PubMed) up to January 2025. The search strategy included the string “(Merkel cell carcinoma AND (bone OR marrow))”. Results: A total of 1133 (69.3% male and 30.7% female) patients diagnosed with advanced MCC were collected. The median (IQR) age at diagnosis was 67.5 (12.65) years old. Overall, 201 (20.8%) cases of bone and/or bone marrow metastases were identified and linked to a primary known MCC in 75.7% of cases. Bone metastases (BMs) appear as the third most common metastatic site, following the liver (second) and lymph nodes (first). They show mixed biological and radiological behavior, with a marked preference for the axial skeleton over the appendicular one. Addressing the characteristics of metastatic bone disease, neurological symptoms were the most documented, whereas bone marrow involvement and leukemic spread seemed to be primarily related to immunosuppression. Multimodal treatment strategies, including platinum-based chemotherapy and radiotherapy, were the primary approaches adopted, reflecting therapeutic practices from the pre-immunotherapy era. Conclusions: The pattern of metastatic spread in MCC differs among studies, with the bones resulting as the third most common site of distant spread. Excluding head and neck MCC, which seems to be more regularly associated with liver metastases, the relationship between the primary tumor site and the development of bone or bone marrow metastases appears inconsistent. Overall, BMs mostly correlated with advanced MCC stages and poorer survival outcomes, with a median overall survival (OS) of 8 months (range 12.75–4). The integration of international guidelines, evolving evidence from clinical trials, and the expanding role of immune checkpoint inhibitors (ICIs) will contribute to improving systemic disease control and enhance patient care.

## Simple Summary

Merkel cell carcinoma (MCC) is a rare and aggressive neuroendocrine skin cancer, known for its high recurrence rate and metastatic potential. Bone metastases have been identified as the fourth or even the third most common site of distant spread. Despite growing awareness of bone and bone marrow involvement in MCC, current research lacks a comprehensive focus on their biological and radiological behavior, the pattern of metastatic spread, and related clinical, demographic, and treatment profiles. This review aims to provide a comprehensive overview of the current evidence regarding MCC, with a focus on the characteristics and impact of bone and bone marrow metastases.

## 1. Introduction

Merkel cell carcinoma (MCC) is a rare yet highly aggressive neuroendocrine skin tumor that predominantly affects elderly men, with the head and extremities being the most common sites of occurrence [1]. In recent years, notable advancements have been achieved in the diagnosis and management of MCC, particularly through the introduction of immunotherapy for locally advanced, inoperable, and metastatic disease [2].

Clinical practice is further guided by several guidelines, which aim to standardize care for MCC patients [2,3,4]. Despite these advancements, the overall prognosis remains historically poor according to pre-immunotherapy data, with a 5-year relative survival rate of 65% across all surveillance, epidemiology, and end results (SEER) stages combined [5]. Moreover, MCC is associated with a high incidence of local recurrence, particularly within the first 2–3 years following the primary tumor excision. Regional nodal metastases develop in 40–50% of patients, while approximately 33% of them experience distant secondaries affecting different anatomical sites [4]. Among these, bone metastases (BMs) have been identified as the fourth [6] or even the third [7] most common site of distant spread.

Nevertheless, despite the increasing recognition of BMs and bone marrow involvement in MCC, the current literature lacks a comprehensive focus on their biological and radiological behavior, the patterns of metastatic spread, and related clinical, demographic, and treatment profiles. This review seeks to offer a comprehensive summary of the latest evidence on MCC epidemiology, etiology, diagnosis, and management, with a focus on the characteristics and impact of bone and bone marrow metastases. It is important to note that most reviewed data, including survival outcomes and patterns of disease progression, stem from studies conducted before the advent of immunotherapy. As a result, current expectations may not fully reflect the impact of recent therapeutic advancements, particularly the introduction of immune checkpoint inhibitors (ICIs).

## 2. Materials and Methods

A comprehensive review was conducted from Medline database (via PubMed) up to January 2025. To address our focus, the search strategy included the string “(Merkel cell carcinoma AND (bone OR marrow))”, which returned 150 articles. Inclusion criteria were (a) studies involving patients diagnosed and treated for metastatic Merkel cell carcinoma, and (b) explicit reporting of bone and/or bone marrow involvement. The exclusion criteria included (a) studies limited to localized Merkel cell carcinoma, and (b) articles not written in English. Additionally, relevant keywords were used in different combinations for free-hand search, and the bibliography of selected articles was reviewed.

## 3. Epidemiology of MCC

Determining precise global incidence rates for Merkel cell carcinoma remains challenging due to geographic and demographic variability, as well as the absence of comprehensive global epidemiological studies. However, between 2000 and 2013, the number of MCC diagnoses increases by 95%, almost doubling the 57% rise in melanoma cases during the same period [8].

In the United States, data from the SEER registry indicate an MCC incidence rate of 0.7 cases per 100,000 person-years in 2013, corresponding to approximately 2488 cases annually [8]. Globally, the incidence of MCC is estimated at 0.6 cases per 100,000 people per year, based on data derived from individual regional reports and isolated case series (9). Notably, countries in the southern hemisphere, such as Australia (2.5 per 100,000) and New Zealand (0.96 per 100,000), report the highest incidence rates, significantly exceeding those observed in the northern hemisphere [9,10]. Conversely, countries such as Norway, Denmark, and Japan demonstrate relatively stable incidence rates over time, representing exceptions to the overall global upward trend in MCC incidence [11].

In addition to geographic and demographic variability, age represents a key epidemiological factor in MCC. Incidence rates increase markedly with age, peaking between 70 and 80 years [11]. Indeed, MCC is relatively rare in younger individuals with only 0.07% of cases occurring in those under 30 years of age [11]. Notably, when affecting young adults, MCC is often diagnosed at more advanced stages [12].

Based on these data, the trend of incidence is a matter of debate. However, several key factors should be considered to understand the phenomenon, combining demographic shifts with environmental exposures and improved diagnostic accuracy: the aging population [10], the ultraviolet (UV) exposure rates [13], the increasing MCC awareness among healthcare providers and patients, the use of non-invasive diagnostic tools (e.g., dermoscopy, reflectance confocal microscopy) [14,15], the greater efficacy and reliance on immunosuppressive therapies [16,17].

## 4. Etiology and Risk Factors for MCC

### 4.1. Two (Viral- and UV-Related) Driving Mechanisms for MCC Onset

Similarly to other cutaneous malignancies, the pathogenesis of Merkel cell carcinoma is likely the result of a complex interaction between genetic, molecular, and environmental factors. Nevertheless, MCC recognizes two main determinants of pathogenesis: viral and UV-exposure-related driving mechanisms.

In 2008, researchers identified the integration of a mutated Merkel cell polyomavirus (MCPyV) genome as a key causative factor in the majority of MCC cases. MCPyV is a circular, double-stranded DNA virus, believed to be acquired during childhood, as evidenced by the widespread presence of antibodies against its major capsid protein, VP1, in the general population. The virus expresses two transforming antigens, small T antigen (sTAg) and truncated large T antigen (LTAg), which contribute to tumorigenesis [18]. sTAg facilitates the recruitment of MYCL to the EP400 complex, leading to downstream effects such as LSD1 upregulation and tumor protein 53 (TP53) inactivation; LTAg promotes the sequestration of retinoblastoma 1 protein (pRb1), resulting in the release and activation of E2F transcription factors, which activate genes involved in DNA replication and cell division [19].

Despite this virus’s widespread occurrence, primary MCPyV infection is typically asymptomatic, with only a small fraction of individuals developing MCC over time [20]. Therefore, MCC is generally classified as MCPyV-positive or -negative cancer [21].

*MCPyV-positive MCC* represents a pure neuroendocrine carcinoma characterized by the expression of viral oncoproteins, a low tumor mutational burden (TMB) [22] and a relatively favorable prognosis compared to the negative counterpart [23]. Although this variant was thought to arise from dermal fibroblasts or pre/pro B-cells [24,25], the latest evidence suggests a common (follicular) epithelial origin. Conversely, patients with MCC often have a history of other skin cancers, particularly those linked to UV exposure, indicating possible shared etiological factors with these malignancies [26,27,28].

In this regard, approximately 20% of MCC cases are *UV-associated MCPyV-negative*. This subtype of MCC, which was found to be more prevalent in certain geographic regions with high levels of sun exposure [29,30], arises from UV-induced DNA damages and displays characteristic oncogenetic features: high TMB, inactivation of tumor suppressor genes (e.g., Rb1 and TP53) [31,32], and high frequency of mutated NOTCH1 and FAT1. UV exposure is also responsible for the inactivation of genes involved in DNA damage repair, including KMT2A, KMT2C KMT2D, ASXL1, ARID1A, ARID1B, SMARCA4, and in chromatin-modifying pathways, such as ATM, MSH2, BRCA1, BRCA2, and BCOR [33]. Moreover, UV-associated MCPyV-negative MCC has been linked with the activation of JAK-STAT, MAPK (HRAS, NF1) [34] and PI3K pathways (PIK3CA, AKT1, PIK3CG), as well as the receptor tyrosine kinase FGFR2 [29].

Within this molecular landscape, p63 has emerged as a relevant biomarker, particularly in MCPyV-negative MCC. A member of the p53 tumor suppressor family, p63, plays a key role in cell cycle regulation existing in two major isoforms: TAp63, with pro-apoptotic functions, and ΔNp63, which exhibits oncogenic properties [35]. Recent studies have prompted a reevaluation of its role in MCC. Elevated mean p63 H-scores (Histological scores or Histoscores) have been observed particularly in MCPyV-negative MCC and combined MCC/squamous cell carcinoma (SCC) cases [36,37], and have been associated with increased mortality (OR 2.92, 95% CI: 1.66–5.13) [35]. Furthermore, p63 expression, especially the TAp63 isoform, appears to correlate with aberrant p53 expression and TP53 mutations [36], both linked to poor clinical outcomes. However, p63 expression shows high intertumoral variability, regardless of MCPyV status, and may be entirely absent even in MCPyV-negative MCC [36], raising concerns about its reliability as a prognostic biomarker.

Accordingly, MCPyV-negative MCC is generally associated with a poorer prognosis [23], and displays either pure neuroendocrine features or a combination of neuroendocrine and SCC characteristics. These tumors appear to originate from or develop in association with SCC, commonly referred to as combined MCC/SCC [19,22,38,39].

Beyond the prolonged exposure to UV radiation and MCPvV infection as established causal mechanisms, several risk factors for MCC are identified. Among these, immunosuppression (e.g., HIV-positive or AIDS patients, organ transplant recipients, or patients undergoing immunosuppressive treatments) [16,40,41], advanced age (over 70 years old) [11], male gender [10,11], fair skin [13], personal history of other skin cancers are the main recognized.

### 4.2. The Debate of MCC Cell of Origin

The identification of the two driving mechanisms in Merkel cell carcinoma pathogenesis has led to a reassessment of its cellular origin, which was originally believed to be the epidermal Merkel cells, from which the tumor receives its name.

Several alternative cells of origin for MCC have been proposed [42,43,44,45] (Table 1). Among these, the epithelial (follicular) lineage has shown robust support for tumorigenesis in recent preclinical models [46].

Merkel cells are highly specialized epithelial cells that function as mechanoreceptors, located in the basal layer of the epidermis and the external part of the hair follicle. Both Merkel cells and MCC share several features. Firstly, both cell types exhibit high expression of the ion channel Piezo2, a protein that facilitates the conversion of mechanical stimuli into electrical signals [47,48]. Secondly, the differentiation of Merkel cells is driven by the expression of a specific transcription factor known as atonal homolog 1 (ATOH1) [49], which is also represented in MCC [50]. Additionally, immunohistochemical studies have shown that both Merkel cells and MCC express common markers, such as cytokeratin (CK) 20, and neuroendocrine markers, like chromogranin A and synaptophysin (SYP) [25].

However, Merkel cells are primarily post-mitotic cells, thus exhibiting low sensitivity to oncogenic triggers [49]. Moreover, they are placed in the basal layer of the epidermis, while MCC typically affects the dermis and subcutaneous tissues [45]. Furthermore, Merkel cells are mostly represented in the palms and soles [51], whereas MCC predominantly occurs in sun-exposed areas, such as the head and neck or limbs [10,42]. Lastly, no reports of Merkel cells being directly infected by Merkel cell polyomavirus have been described [52].

The similarities between Merkel cells and MCC may reflect the acquisition of a neuroendocrine phenotype by different cell types during the oncogenic transformation leading to MCC [53]. Indeed, both sun exposure and virus-induced oncogenic triggers may act on shared molecular pathways, notably involving the loss of the Rb protein. In this sense, the sequestration of the tumor suppressor Rb by MCPyV is a critical step in the pathogenesis of MCPyV-positive MCC [31,54]. The Rb expression is usually retained, but functionally inactivated: LTAg binds and functionally inhibits pRb1 without deleting or mutating the RB1 gene, making the Rb protein still detectable by immunohistochemistry, but functionally inactive. Alternatively, the loss of Rb expression in MCPyV-negative MCC tumors is attributed to somatic mutations [31,32]. Supporting this concept, studies involving other cancer types, such as colorectal neuroendocrine carcinoma and small cell lung carcinoma (SCLC), have demonstrated that the loss of Rb function contributes to the development of a neuroendocrine phenotype [55,56,57]. Furthermore, the Rb inactivation may lead to increased expression of ATOH1, thus inducing Merkel cell differentiation [58].

Based on these findings, alternative origins for MCC cells have been considered, including epithelial non-Merkel cells, fibroblastic cells, and B-cell lineages [25,59,60,61].

Evidence from MCPyV-negative MCC cases supports these tumors arising from epidermal progenitor cells exposed to chronic UV damage [62]. Notably, the UV mutation signature is characterized by specific types of mutations, particularly C to T transitions, frequently involving key tumor suppressor genes like TP53 and Rb1. Dual inactivation of these genes is documented in SCLC, driving both transformation and neuroendocrine differentiation in epithelial cells [63,64].

This oncogenic pathway may be relevant to combined MCC/SCC tumors, which are generally MCPyV-negative. Both MCC and SCC share a high TMB, marked by a predominant UV-signature mutation profile. Importantly, the increased allelic frequencies of mutations shared between the SCC and MCC components indicate that the MCC may have developed through clonal expansion of an SCC cell that previously accumulated these genetic alterations [19]. Afterward, the shift to the neuroendocrine phenotype seems to be linked to the loss of pRb function and the increasing expression of Merkel cell genes (e.g., SOX2) [38,65].

Complicating the current understanding, DNA methylation profiling has provided evidence supporting a keratinocytic origin for MCPyV-positive MCC. This epigenetic analysis revealed similarities between virus-positive and virus-negative MCC cell lines, pointing toward a shared epithelial lineage [66]. The absence of a UV-induced mutational signature in MCPyV-positive cases may reflect an origin from cells located in sun-protected niches, such as the hair follicles.

This hypothesis is further supported by phenotypic similarities between Merkel cell progenitors and trichoblastoma, a follicular epithelial neoplasm. Notably, a rare, combined tumor comprising trichoblastoma and MCPyV-positive MCC exhibited shared somatic mutations, with MCPyV integration detected exclusively in the MCC component. These findings suggest that viral transformation likely occurred within a follicular epithelial progenitor cell [67].

Moreover, recent work by Verhaegen et al. provided experimental support for a follicular epithelial origin of MCC and highlighted the critical role of TP53 inactivation in its development. Using a preclinical murine model, the authors demonstrated that the expression of MCPyV T antigens under the control of ATOH1 in KRT5-expressing epidermal cells was sufficient to initiate the formation of MCC-like lesions [46]. However, only upon targeted deletion of TP53 did the mice develop skin tumors with classic MCC histopathology, localized to the dermis without clear connection to the epidermis or hair follicles, and with characteristic immunophenotypic features, including dot-like expression of KRT8, SOX2, ISL1, insulinoma-associated protein 1 (INSM1), and POU3F2 [46]. Unlike genomic inactivation, TP53 in the human setting may be functionally suppressed through MCPyV sTAg-mediated upregulation of MDM2, further supporting the follicular genesis of MCPyV-positive MCC. Nevertheless, the specific cell type in which MCPyV integration occurs is still unclear.

Even so, although recent evidence mostly supports the epidermal (follicular) origin of MCC, several observations point toward a possible non-epithelial origin for this neoplasia: the absence of connection between the tumor cells and the epidermis, the lack of a UV-mutational signature and the low TMB of MCPyV-positive tumors [44]. In this context, dermal mesenchymal cells, such as fibroblast stem cells, are proposed as candidates due to their deep dermal location. This hypothesis is further supported by studies showing that dermal fibroblasts can sustain the MCPyV life cycle in vitro [68,69]. Moreover, fibroblasts have the potential to be reprogrammed into pluripotent cells [70], suggesting they could adopt a Merkel cell phenotype. However, this theoretical concept still needs to be experimentally validated.

Beyond the absence of an epidermal connection, MCC may express B cell markers, such as TdT and PAX5, with some MCPyV-positive MCC cases exhibiting immunoglobulin (Ig) rearrangement. These findings indicate that MCPyV-positive MCC may originate from pre-/pro-B cells [71]. Furthermore, both MCPyV-positive and -negative MCCs often share phenotypic similarities with B-cell neoplasia, and MCPyV can integrate itself into hematopoietic cells, potentially guiding the transformation of B cells. To date, the failure to acquire a Merkel cell phenotype in these instances argues against B-cell origin of MCC [24].

## 5. Clinical Features and Diagnosis of MCC

The diagnosis of Merkel cell carcinoma is established through a comprehensive approach that includes clinical assessment, noninvasive imaging, and histopathological analysis with immunohistochemistry.

From a clinical perspective, MCC typically presents as a pink or red-violaceous, painless, firm, rapidly growing nodule or plaque, this last one theoretically more detectable in the early stages of the disease [1]. Four distinct clinical presentations have been proposed and described as representative of MCC: pinkish plaque, cherry red nodular, ulcerated erythematous nodular, and hyperkeratotic nodular MCC [72]. Interestingly, while the first three clinical findings were histologically consistent with pure MCC, the final one was identified as combined MCC/SCC [72]. Compared to pure MCPyV-positive MCC, the coexistence of MCC with SCC is relatively rare, more frequently observed in immunosuppressed individuals and associated with poorer clinical outcomes [73,74]. MCC may also coexist with Bowen’s disease (BD)/in situ SCC, presenting more commonly in females as a rapidly growing solitary nodule arising on a red-brown plaque, typically located on the face [75]. However, the apparent female predominance is not consistently observed across studies [76]. Given the paucity of comparative analyses between combined and pure MCC, further international collaborative efforts are needed to generate high-quality evidence and validate these preliminary observations [39].

Although MCC is commonly detected in sun-exposed areas, it can also rarely arise from the oral mucosa and/or lips, which are of dermatological interest as potential primary tumor sites. This has been reported in both adults [77,78,79,80,81,82] and young individuals, starting from the age of 14 [83,84,85]. In these cases, MCC typically presents as an ulceroproliferative or pinkish red, rapidly growing nodule, especially when located at the vermillion border or labial semi-/mucosa [81]. Furthermore, cases of MCC involving the penile or scrotal region have been documented less frequently [86,87] compared to those occurring in the vulvar region [88,89]. Pain and perilesional erythema have also been noted as additional characteristics for these specific locations. A detailed analysis and data collection on these special site locations for MCC are beyond the scope of this review.

Along with clinical evaluation, dermoscopy and reflectance confocal microscopy (RCM) represent two non-invasive imaging techniques that have proven to be highly valuable in diagnosing MCC [90,91]. The primary dermoscopic features of MCC include a variably focused and dilated polymorphous vessels set on a homogeneous pinkish, milky red structureless background, together with shiny or not-shiny white areas [72,91].

Different from BD and its glomerular and/or dotted vessels at dermoscopy [90], in MCC the dermoscopic vascular pattern is characterized by irregular linear vessels, either alone or in combination with glomerular or arborizing vessels supporting the differential diagnosis [72].

In RCM, MCC displays aggregates of hyporeflective small cells bordered by fibrotic linear septae, which have been previously reported as highly suggestive of the disease [91] in support of a clinical and dermoscopic suspicion of MCC [72]. Additionally, larger polymorphic hyper-reflective cells, likely representing highly proliferative cells, may also be observed [91].

Given that, the final MCC diagnosis relies on the histological examination revealing a tumor that diffusely infiltrates the dermis/hypodermis and consists of small, round, and blue cells, as revealed by hematoxylin-eosin staining [92,93,94,95,96,97,98]. These cells characteristically display a fine stippled chromatin and absent nucleoli, and they express neuroendocrine differentiation markers, including chromogranin A, SYP, cluster of differentiation 56 (CD56), neuron-specific enolase (NSE), neurofilaments (NF), and INSM1 [92,93,94,95,96,97,98].

Notably, MCC cells express various CKs, including CK20 with a characteristic dot-like paranuclear, CK8, CK18, and CK19, and being almost invariably negative for CK7 [92,93,94,95,96,97,98]. However, a small minority of MCC cases (less than 10%), most of which are UV-induced and not associated with MCPyV, are completely negative for CK20, which may be a diagnostic pitfall [99]. Additionally, MCC is typically negative for thyroid transcription factor 1 (TTF-1), leukocyte common antigen/cluster of differentiation 45 (LCA/CD45), other lymphocytic/lymphoblastic markers (CD20, CD3, and TdT), S100, SOX10, other melanocytic markers (Melan A/Mart-1, HMB45), vimentin, CK7, CD99, and sal-like protein 4 (SALL4) [92,93,94,95,96,97,98].

Thus, an immunohistochemical panel showing positivity for neuroendocrine markers, NF, CK20 (paranuclear dot-like pattern) and negativity for TTF-1, CK7, melanocytic and lymphocytic/lymphoblastic markers offer high sensitivity and specificity rates for differentiating MCC from the common histologic mimickers, such as SCLC, neuroblastoma, Ewing sarcoma, melanoma, lymphomas, and germ cell tumors (Table 2) [92,93,94,95,96,97,98]. Nonetheless, MCC may rarely stain for TTF-1, CK7, and other lineage markers, as well as being negative for CK20 in a minority of cases [92,93,94,95,96,97,98].

As a result, its diagnosis may be challenging in a subgroup of patients and require caution when interpreting the staining patterns and integration of a patient’s clinical history and clinical–radiological exams. For this reason, several studies have tested various and new immunohistochemical markers in MCC, such as SATB2, PRAME, ALK, EZH2, Rb, SOX2, and MCPyV LTAg [65,100,101,102,103,104,105].

These studies have clarified multiple aspects of MCC pathogenesis, including theories of histogenesis and the distinctions between MCPyV-associated and UV-induced subtypes. Moreover, they have contributed to the development of novel diagnostic and therapeutic tools for this aggressive tumor [65,100,101,102,104,105].

In parallel, other studies focused on neuroendocrine carcinomas and high-grade neuroendocrine tumors of specific sites to verify whether specific “site-markers” could help to distinguish them from MCC (e.g., CDX2 for gastrointestinal tumors, GATA3 for urogenital and breast tumors, and NKX3.1 for prostate tumors) [106,107,108,109]. Overall, these studies have enabled the identification of immunohistochemical panels that can help differentiate MCC from its mimickers, especially in specific diagnostic scenarios (e.g., prostatic neuroendocrine carcinomas that express CK20 and/or MCC with aberrant expression of other lineage markers) (Table 2) [65,92,93,94,95,96,97,98,100,101,102,103,104,105].

**Figure 1:**
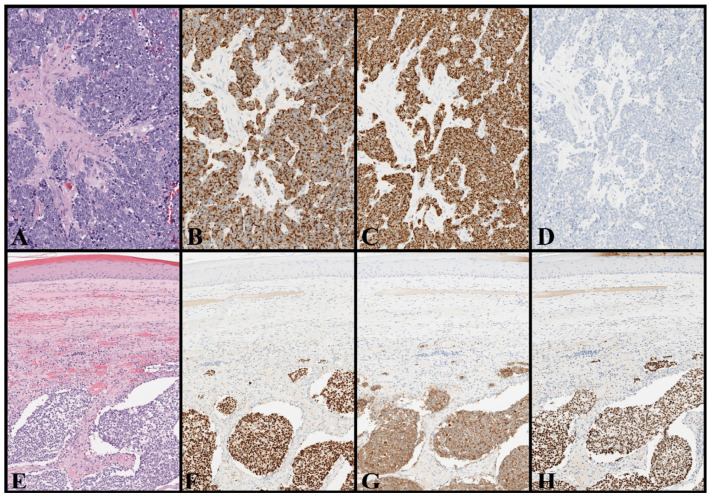
Histopathological and immunohistochemical features of Merkel cell carcinoma. MCC ((**A**), H&E original magnification ×200) with diffuse chromogranin A ((**B**) original magnification ×200) and CK20 ((**C**) original magnification ×200) positivity, but completely negative for CK7 ((**D**) original magnification ×200). MCC ((**E**), H&E, original magnification ×150) exhibits strong and diffuse INSM1 ((**F**) original magnification ×150), synaptophysin ((**G**) original magnification ×150), and SATB2 ((**H**) original magnification ×150) stain.

**Figure 2:**
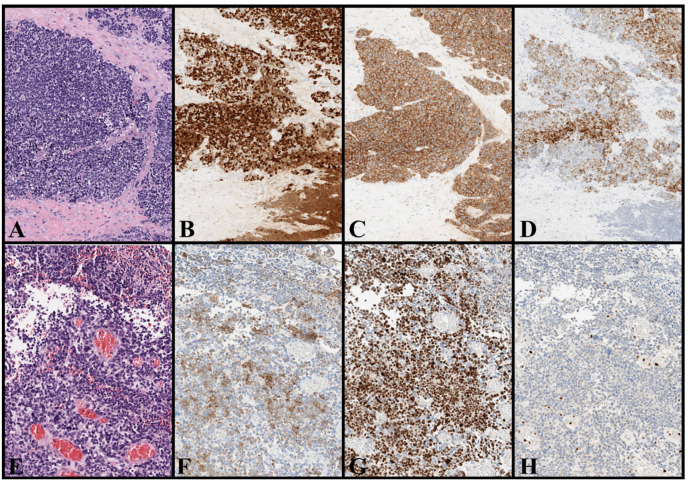
Histologic and immunohistochemical features supporting the differential diagnosis of small cell carcinomas of the lung (SCLC) and bladder. Small cell lung cancer ((**A**), H&E original magnification ×200) with diffuse INSM1 ((**B**) original magnification ×200) and synaptophysin ((**C**) original magnification ×200) positivity, but patchy/focal stain for TTF-1 ((**D**) original magnification ×200). Small cell bladder cancer ((**E**), H&E, original magnification x 180) exhibits patchy/focal stain for synaptophysin ((**F**) original magnification ×150), diffuse positivity for GATA3 ((**G**) original magnification ×150), and complete loss of Rb ((**H**) original magnification ×150) stain.

Illustrative examples of MCC and potential histologic mimickers are shown in Figure 1 and Figure 2. No histological markers can selectively differentiate between MCPyV- and UV-induced MCC, but a positive staining for MCPyV LTAg strongly indicates MCPyV-induced MCC [105]. Nevertheless, negative staining does not definitively rule out the possibility of MCPyV involvement and several molecular and epigenetic markers have been tested to aid in differentiation [53,105,110,111,112]. It is important to note that these two different forms of MCC may have common pathogenic pathways and several genetic and epigenetic overlaps [53,105,110,111,112]. Therefore, in specific cases, only molecular demonstration of MCPyV (whether integrated into tumor DNA or not) can solve this diagnostic dilemma [97,98,105,111,112].

## 6. Staging System (AJCC Eighth Edition) and Prognostic Factors

Staging of Merkel Cell Carcinoma involves the TNM system, evaluating the size and extent of the primary tumor (T), the involvement of regional lymph nodes (N), and the presence of distant metastases (M) [113]. The American Joint Committee on Cancer (AJCC) classification (eighth edition) refines this by distinguishing lymph node involvement into clinical (N) and pathological (pN) stages, based on whether lymph nodes are assessed through physical examination or histopathological evaluation [113]. According to this system (Table 3), MCC is classified as follows: stages I and II for skin-limited disease; stage III for regional lymph node involvement or an undetectable primary tumor; and stage IV for distant metastatic disease beyond regional lymph nodes [113].

Clinical staging relies on physical examination, lymph node palpation, and imaging studies. Routine baseline imaging is consistently recommended to confirm that MCC is localized, with clinically node-negative (cN0) and no evidence of distant disease [2]. Several studies demonstrated that whole-body 2-deoxy-2-[^18^F] fluoro-D-glucose positron emission tomography/computed tomography ([^18^F] FDG-PET/CT) is a valuable tool for initial staging in cN0 patients, given its high sensitivity in detecting occult metastatic disease [114]. Furthermore, FDG-PET/CT plays a critical role in disease re-staging. Contrast-enhanced CT of the chest, abdomen, and pelvis, along with the neck if the primary tumor is in the head or neck region, represents an acceptable alternative for initial imaging assessment [115]. Imaging can also aid in distinguishing primary cutaneous MCC from cutaneous metastases of noncutaneous neuroendocrine carcinomas, such as SCLC, particularly in cases where immunohistochemical markers are atypical (e.g., CK20-negative and/or TTF-1–positive).

Sentinel lymph node biopsy (SLNB) represents the most reliable method for detecting subclinical nodal involvement, utilizing a specific immunohistochemical panel [3]. Pathological staging involves microscopic examination of tissue samples obtained through lymph node biopsies, organ biopsies, or needle biopsies [94,113,116].

Survival in MCC is mainly determined by the stage at diagnosis. The 5-year overall survival (OS) rate drops significantly as the stage advances, from 62.8% at stage I to 13.5% at stage IV (AJCC eighth edition) according to pre-immunotherapy data [117,118]. Nevertheless, a recent cohort study interestingly found that only 65% of deaths were directly attributed to MCC-related causes [119]. As with survival data, in terms of prognosis, MCC is also characterized by high recurrence rates: local recurrence (27–60%), regional lymph node involvement (45–91%), and distant metastasis (13–52%) [113,120,121].

Integrating current evidence, MCC patients are considered as *high-risk for recurrence* if they exhibit one or more modified adverse risk factors (mARF), including tumor size ≥2 cm (or >1 cm per NCCN guidelines v1.2024), chronic immunosuppression (e.g., HIV, chronic lymphocytic leukemia, or solid organ transplant), head and neck primary sites, lymphovascular invasion, pathologically positive lymph nodes, or incomplete lymph node evaluation [2,3,4,122,123].

Several tumor markers, such as Rb protein expression, intratumoral CD8+ T-lymphocyte infiltration, and MCPyV LTAg expression, have also shown potential as positive prognostic indicators, although further validation is required [3].

Moreover, serologic testing for MCPyV oncoprotein antibodies should be considered as part of the initial prognostic assessment. Seropositive patients may benefit from longitudinal monitoring of antibody titers, which could aid in detecting recurrence and potentially reduce the reliance on frequent imaging [124]. Conversely, seronegative patients face a 42% higher recurrence risk and necessitate a closer surveillance [124].

Due to the rarity of MCC, the understanding of metastatic patterns and prognosis at stage IV remains incomplete, with data showing considerable variability. Patients with bone or liver metastases report significantly worse OS (*p* < 0.01) and an increased risk of Merkel-specific death (HR: 3.06 for bone metastases and HR: 2.09 for liver metastases, *p* < 0.001) [7]. However, another study also conducted prior to the introduction of immunotherapy found that while liver and brain metastases were significantly associated with poorer disease-specific survival (DSS), bone metastases did not demonstrate a similar correlation [125]. Additionally, patients with metastases to the bone, liver, and distant lymph nodes seemed to also have a higher risk of regional lymph node involvement [122].

Excluding the following articles due to the lack of individual data [6,125,126,127,128,129,130] (Table 4), the median OS for bone and bone marrow metastatic MCC was 8 months (range 12.75–4). This likely reflects the predominance of data generated before the introduction of systemic immunotherapy. As the role of ICIs continues to evolve, their contribution to improved systemic disease control and patient outcomes is increasingly evident. Nonetheless, further well-designed studies are required to provide definitive and comprehensive evaluations.

## 7. Bone and Bone Marrow Metastases in MCC

### 7.1. Type of Bone Metastases

Bone metastases (BMs) can be classified in two types according to histopathology: osteoblastic or bone-forming BMs, as reported in prostate cancer and breast cancer; osteolytic or bone-destructive BMs, as metastases from kidney, thyroid, lung cancers, multiple myeloma; and mixed BMs, namely the combination of both osteoblastic and osteolytic processes (as reported less often in breast cancer) [167].

Considering the literature on the biological behavior of these different pathological bone secondaries, diversification partially influences prognosis since osteolytic lesions are often more aggressive and generally show quicker progression compared to the sclerotic metastases [167,168]. Additionally, tumor cell proliferation within bone marrow precedes bone destruction, making bone damage a relatively delayed feature of BMs [168,169], and explaining the variable development of bone pain and fractures.

There is limited data on the nature of BMs in Merkel cell carcinoma. The evidence derived from imaging studies suggests a major mixed behavior, as demonstrated by radiopaque/hyperdense (when osteoblastic) or radiolucent/hypodense (when osteolytic) signals from radiography (X-ray) or computed tomography (CT) scans, respectively [170]. Cases of intracranial metastasis through [171] or without [131] bone destructions have been reported, although arterial or venous spread represents the most common form of dissemination [131]. The specific venous drainage and veno-lymphatic anastomoses generated by MCC neoplastic clones may explain the evidence.

### 7.2. Pattern of Metastatic Spread and Association Between Primary MCC and BMs

Common distant metastatic sites include non-regional LNs (41%), skin/soft tissue (25%), liver (23%), bone (21%), pancreas (8%), lung (7%), and brain (5%) (percentages exceeded 100% because some patients had metastatic disease involving multiple sites) [6]. However, the reported patterns of metastatic spread in the literature vary across different studies. In 2024, Kim et al. analyzed 151 patients who had received treatment for MCC and examined the relationship between the primary tumor site and distant metastases. They found that after a median follow-up of 11 months, 58.9% of patients had a single metastatic site, while 41.1% developed multiple. The most common metastases were distant LNs (62.3%), followed by skin/soft tissues (26.5%) and bones (26.5%) [7].

Some authors reported different data about the most common distant metastatic sites in MCC. BMs are typically preceded by other involvements such as non-regional LNs (the most frequent), skin, and lung [117] or abdominal organs [121]. Nevertheless, non-regional LNs are not always the primary sites affected by distant metastases. Gonzalez et al. reported that the most common metastatic sites were the liver (39.3%), followed by distant LNs (38.3%), bone (27.7%), and lung (21.9%) [122]. Similarly, Xia et al. found liver metastases to be the most common (13.5%), followed by bone (11.3%) and lung (8.4%) secondaries [126].

Regarding the association between the primary MCC and BMs, notably, patients with upper limb/shoulder primaries were less likely to develop distant LNs or liver metastases (*p* = 0.02 and *p* = 0.04), while those with head and neck primaries were more likely to develop liver metastases (*p* < 0.01) [7].

This observation was supported by Maloney et al., who demonstrated that head and neck primary MCC was associated with liver metastases (*p* = 0.0003), in contrast to primary tumors of the lower limbs [125]. These findings regarding the likelihood of liver metastases in MCC are consistent with previously published data, which show a higher incidence involving the head/neck primary tumors (43% of 58 patients) compared to lower limb primaries (5% of 39 patients; *p* < 0.0001) [6].

On the other hand, patients with trunk primary MCCs exhibited higher rates of positive lymph nodes and seemed more prone to developing BMs (*p* = 0.0049) [172]. Nevertheless, these data are not fully corroborated, as Kim et al. found bone metastases to be more frequently associated with head and neck (37.5%) or upper limb primaries (22.5%) compared to trunk ones (17.5%) [7].

In this review, we summarized the prevalence of single metastatic localizations from SEER databases and retrospective mono-/multi-centric studies, collecting a total of 967 metastatic events at presentation or during follow up reports. In decreasing order of prevalence, the pattern of metastatic spread was as follows: LNs (27.8%, n = 269), liver (21.2%, n = 205), bone (19%, n = 184), lung (10.5%, n = 102), skin and subcutaneous tissue (8.6%, n = 83), brain (3.1%, n = 30), gastric (2.3%, n = 23), spinal cord (2.2%, n = 21), bone marrow (1.8%, n = 17), pancreas (1.4%, n = 14), testis (1.2%, n = 12), retroperitoneum/thyroid gland (0.4%, n = 3 each), and heart (0.1%, n = 1) [6,7,123,125,126,127,128,129,130,131,132,133,134,135,136,137,138,139,140,141,142,143,144,145,146,147,148,149,150,151,152,153,154,155,156,157,158,159,160,161,162,163,164,165,173,174,175,176,177,178,179,180]. Overall, BMs and bone marrow involvement in MCC are linked to advanced stages [130,150,151] and associated with poorer survival outcomes [122,132].

### 7.3. Clinical and Demographic Data

In total, 44 articles retrieved out of 150 reported bone/bone marrow as a metastatic site (Table 4). A total of 1133 (69.3% male and 30.7% female) patients diagnosed with advanced MCC were surveyed. The median (IQR) age at diagnosis was 67.5 (12.65) years old. A total of 201 (20.8%%) cases of bone and/or bone marrow metastases were identified and linked to a primary known and unknown MCC in 75.7% and 24.3% of cases, respectively. Sometimes the nature of the primary MCC was not otherwise specified [125,129].

Except for one instance [127], in single-case reports of bone/bone marrow MCC metastases specifically detailing primary tumor sites (n = 30) [127,128,130,131,133,134,135,136,137,138,139,140,141,142,143,144,145,146,147,148,149,150,151,152,153,154,156,157,158,159,160,161,162,163,164,165,166], the anatomical distribution of the primary MCC aligned with typical patterns described in the literature to date for this neuroendocrine skin tumor, specifically the head and neck (12/28), trunk (8/28), upper (6/28) and lower arms (2/28), and hand (1/28). Notably, an extremely rare case of MCC arising on the vulva was reported [130].

Considering bone localization among all cases reviewed, the specific type of skeletal involvement other than “bone involvement” was documented in 37 patients, with a notable predilection for the axial skeleton (35 cases) [127,128,130,131,133,134,135,136,137,138,139,140,141,142,143,144,145,146,147,148,149,150,151,154,156,157,158,159,160,161,162,163,164,165] over the appendicular one (2 cases) [152,153].Additionally, a total of 17 cases of bone marrow involvement have been reported [127,128,130,137,138,140,141,142,143,144,145,146,148,164,165]. In instances of extramedullary intraspinal MCC metastases, epidural involvement [134,149,150,151,157,158,159] was more frequently observed than intradural spread [130,134,135].

Clinically, bone metastases were mainly asymptomatic. In the subset of symptomatic cases, 10 out of 39 reports (25%), symptoms were location-dependent. Pain was the most frequently reported [151,152,162], followed by neurological manifestations, including seizures [147], weakness/numbness [150,157,159,162], paresthesia [151], balance disturbance [134], and paraplegias following extra-dural spinal masses [149,157]; headaches were also noted [134].

Interestingly, leukemic spread during bone marrow disease was reported and linked to certain forms of immunosuppression, suggesting a potential association. These included patients receiving organ transplantation [139,141,164,165], patients with concomitant autoimmune disorders and treated with immunosuppressant therapies (systemic lupus erythematous [137,142], rheumatoid arthritis [138,146,165], and patients affected by different hematologic conditions (Waldenström macroglobulinemia, plasma cell myeloma, myelodysplastic syndrome, JAK2-positive polycythemia vera, chronic lymphocytic leukemia) [127,128,140,145]. In one case, MCC in a pregnant woman led to a rapidly progressing visceral multi-metastatic disease that proved fatal [162].

### 7.4. Imaging Features of MCC Across Different Diagnostic Techniques

**Figure 3:**
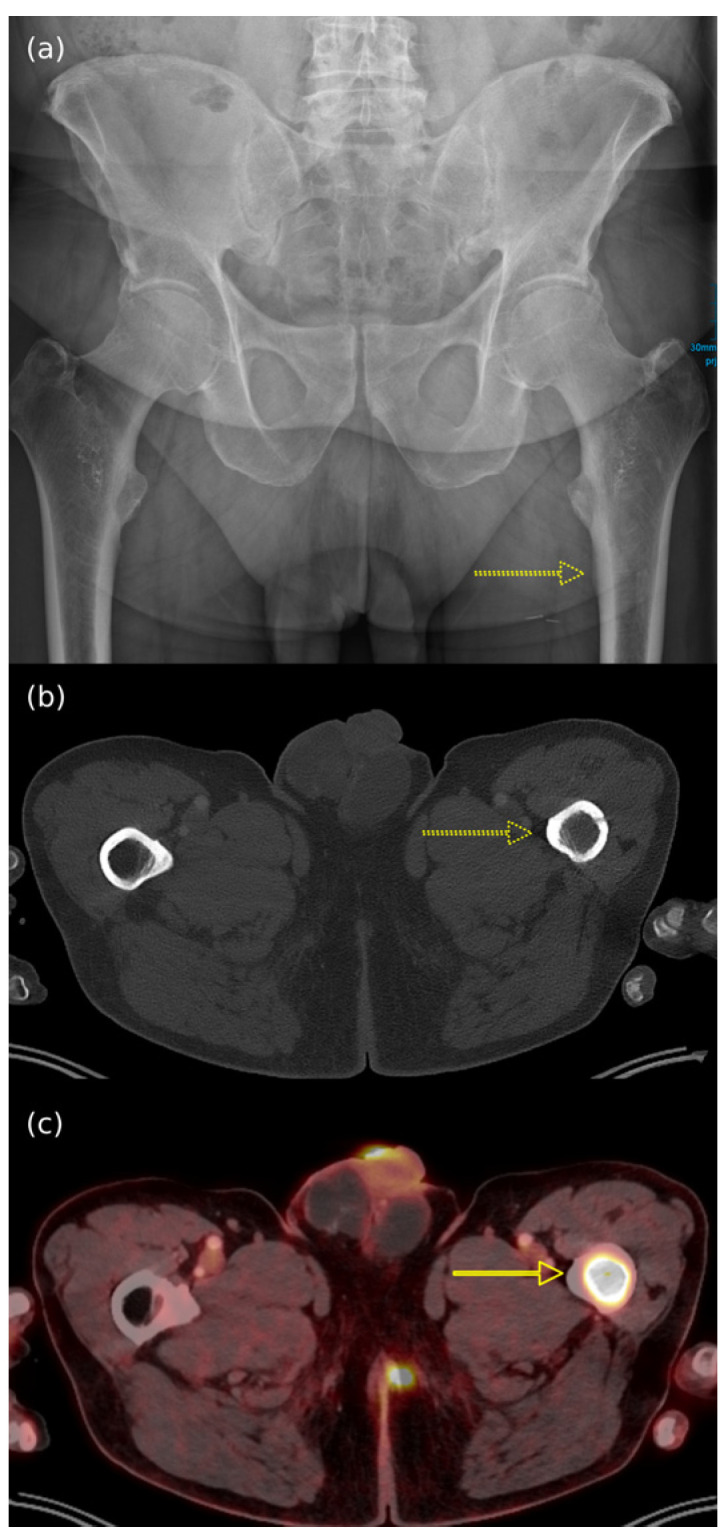
Multimodal imaging of MCC bone metastasis. (**a**,**b**) X-ray and CT images of the left femur showing no structural abnormalities (yellow arrows). (**c**) PET/CT reveals increased metabolic activity in the same region, consistent with bone marrow involvement (yellow arrow).

Imaging represents an essential tool in Merkel cell carcinoma management, from early detection to accurate staging. Whole-body FDG-PET/CT or whole-body contrast-enhanced CT scans are mandatory to assess disease extension [3]. PET/CT scans have been reported as more sensitive than CT alone, resulting in an upstage of the disease (7% of cases, mainly stage I/II to stage III) [115,181,182]; therefore, it should be preferred over CT alone when available [182] (Figure 3). Additionally, 8–14-megahertz regional lymph node ultrasound (US) should be integrated to examine all the main lymph node basins in patients with clinical stage I-II at baseline [3].

To date, the indications about the execution of head/brain imaging differ slightly among the guidelines considered: while ESMO-EURACAN Clinical Practice Guidelines indicated brain imaging for head/neck-located MCC primaries [3], the European-consensus-based interdisciplinary guideline did not routinely recommend it in asymptomatic stage I/II patients [4].

A synthesis of the literature data supports the use of whole-body imaging, such as PET/CT scans, extending to the neck when the primary tumor involves the head or neck region, as part of the baseline assessment. Additionally, brain magnetic resonance imaging (MRI) should be conducted in cases of neurological symptoms or when a direct cranial extension of the tumor is suspected [2,3,4]. In our experience, for elderly patients with head and neck MCC presenting with at least one mARF or a negative SLNB, we typically recommend whole-body PET/CT imaging integrated with head and neck CT or MRI scan.

Primary MCC has nonspecific strictly imaging features [170,183]. Nevertheless, certain suggestive findings can aid in its identification: a cutaneous or subcutaneous firm nodule/mass within or around muscle tissue; necrosis in larger lesions (>2 cm) [170]; significant contrast uptake both for CT and MRI, in accordance with the pathology of fibrovascular separation between clusters of tumor cells and enriched blood sinuses [52].

**Figure 4:**
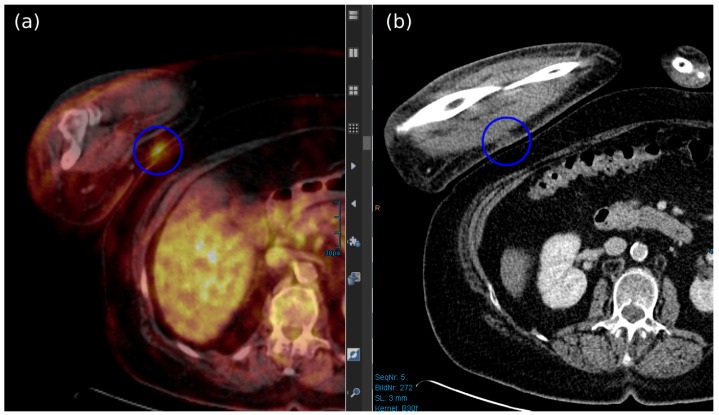
PET/CT and CT imaging of Merkel cell carcinoma. (**a**) PET/CT and (**b**) CT show an FDG–avid subcutaneous nodule on the volar aspect of the right forearm (blue circle), histologically confirmed as MCC.

**Figure 5:**
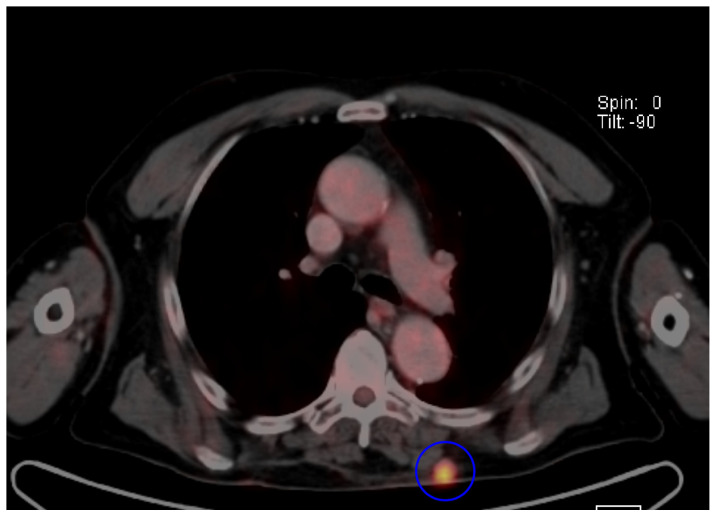
PET/CT imaging of Merkel cell carcinoma. PET/CT scan reveals an FDG–avid subcutaneous nodular lesion on the back (blue circle), confirmed as MCC by histopathological examination of the excised specimen.

Among the different imaging techniques available, primary MCC mainly appears as follows: (a) on US, it shows heterogeneous echogenicity (mainly hypoechoic nodules arising from the dermis/hypodermis), with prominent branching/chaotic vascularity, and occasional perpendicular hypoechoic linear bands resembling “columns of smoke”; (b) on CT, focal skin thickening associated with the cutaneous/subcutaneous nodule appears, with contrast enhancing that is particularly evident for lesions located in the subcutaneous tissue, along with signs of tissue edema (Figure 4b); (c) on MRI, there is hypo- to isointensity on T1-weighted images and either hyperintensity or isointensity on T2-weighted images and STIR sequences; (d) on FDG-PET/CT, the hypermetabolic signal is consistent with a malignant proliferation, particularly for a SUV value of 7.5 ± 3.9 (mean ± SD) [7,52,183,184] (Figure 4a and Figure 5).

A recent umbrella review of meta-analyses aimed to provide updated evidence to guide appropriate referrals for specific radiopharmaceutical PET/CT or PET/MRI in solid cancers. This review reported a sensitivity and specificity of 0.91 (95% CI 0.85–0.95) and 0.93 (95% CI 0.86–0.97), respectively, for nodal staging in MCC using FDG-PET/CT [184]. It also recommended FDG-PET/CT for initial lymph node staging and for cases of nodal or distant metastases from unknown primary MCC. Otherwise, DOTA-peptide imaging, specifically using Gallium-68 (68Ga)-labeled DOTA-peptides (tetraxetan), can be considered in case of FDG-PET/CT negativity [184].

Limited data have been published in the literature regarding imaging features of bone-metastatic MCC. PET/CT, PET/MRI, and CT scans are more effective than MRI alone in detecting bone abnormalities (cortical destruction and/or periosteal reaction) and accurately defining bone signals, as demonstrated in well-known metastatic bone cancers [185]. However, due to its high sensitivity, MRI can be particularly useful for the detection of bone marrow involvement and extraosseous extension of the tumor [151,186]. Additionally, this method is warranted in cases of cord compression from pathologic vertebral body fracture and/or spinal cord oedema (focal high T2 phase) [160]. Nonetheless, using intravenous contrast, T1-weighted MRI with fat saturation (STIR) will show intense uptake in the metastatic body, along with any associated variable focal areas of hypointense necrosis [187].

**Figure 6:**
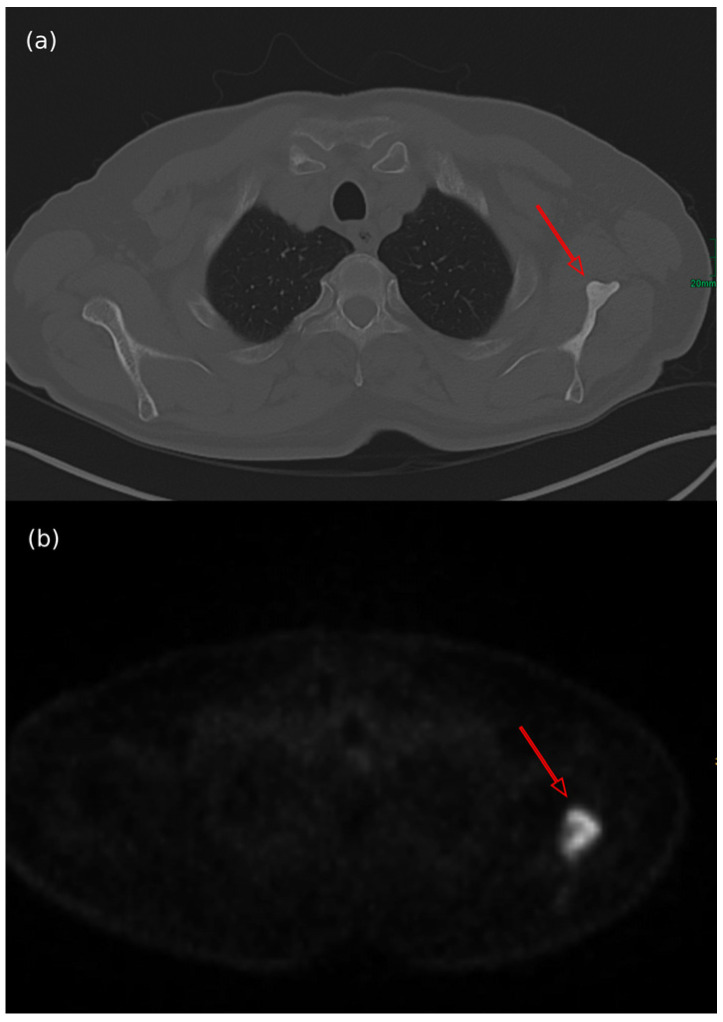
Osteoblastic bone metastasis from Merkel cell carcinoma. (**a**) Axial CT image showing an osteoblastic lesion in the left scapula (red arrow). (**b**) Corresponding PET scan reveals FDG uptake in the same region (red arrow), consistent with metabolically active metastatic disease.

**Figure 7:**
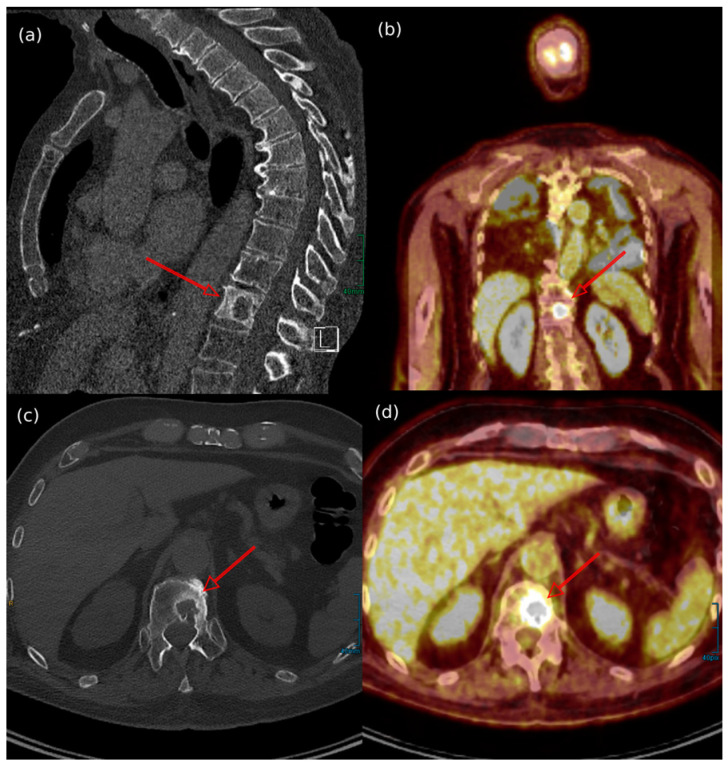
Osteolytic bone metastasis from Merkel cell carcinoma. Sagittal (**a**) and axial (**c**) CT images show an osteolytic lesion in the D10 vertebral body (red arrows). Corresponding coronal (**b**) and axial (**d**) PET/CT images demonstrate FDG uptake in the same lesion (red arrows). No gross epidural involvement is observed in this patient.

As mentioned above, the mixed biologic behavior of BMs in MCC accounts for the variable response to X-ray-based imaging, depending on the degree of absorption [170]. In X-ray or CT scans, while the osteoblastic lesions appear as round/nodular, well-defined, radiodense, or hyperdense bone lesions (Figure 6a), the osteolytic BMs are described as ill-defined, with thinned/absent trabeculae, lucent, or hypodense bone lesions (Figure 7a,c) [153]. When contrast enhancement is performed, hypervascularity during the arterial phase of enhancement explains the signal increase [188].

In FDG-PET/CT, the focal radiotracer uptake in the bones involved is typical (hypermetabolic lesions) (Figure 6b), with osteolytic metastases presenting as photopenic and characterized by increased peripheral activity (Figure 7b,d) [189,190].

**Figure 8:**
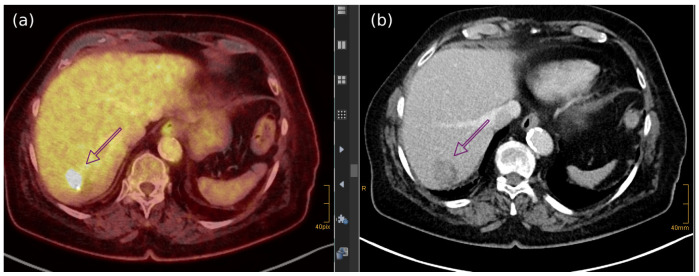
Hepatic metastasis from Merkel cell carcinoma. (**a**) PET/CT image shows an FDG–avid lesion in segment VII of the liver (purple arrow). (**b**) Corresponding CT image reveals approximately 3 cm enhancing nodule with irregular margins, no signs of central necrosis or capsular retraction.

Whole-body PET/MRI has demonstrated superior detection of liver metastases compared to PET/CT, although these data have not yet been tested for MCC [191] (Figure 8).

Among the other imaging techniques adopted to detect BMs and mentioned in the current guidelines, bone scintigraphy is useful and generally used as first-line modality in patients with suspect bone metastases. Using a radioactive substance, typically technetium-99m (Tc-99m) labeled with a bone-seeking compound like methylene diphosphonate (MDP), it provides whole-body imaging; although less specific, it requires far smaller changes in normal-to-abnormal bone for detection compared to plain radiographs [192].

Finally, the overall sensitivity results among the different radiologic techniques are given as follows: plain radiographs have low sensitivity (~50%) compared to bone scintigraphy (80%, range 62–100%), CT (85%, range 71–100%) and MRI (90%, range 82–100%) [192,193,194].

As high-grade neuroendocrine tumors, Merkel cell carcinomas express somatostatin receptors (SSTR), which may be utilized for visualization of disease burden. In this setting, ^111^in-pentetreotide scintigraphy (OctreoScan) has been mainly used in the diagnostic workup of MCC, though with variable accuracy [195].

More recently, SSTR-PET using radiotracers [^68^Ga]DOTA-D-Phe^1^-Tyr^3^-octreotide (DOTATOC) or -octreotate (DOTATATE) has shown high sensitivity for imaging bone, soft tissue and brain metastases, particularly when in combination with CT [196].

Real-world studies have demonstrated its clinical utility in detecting lymph node metastases [196] and, in some cases, revealing a more extensive tumor burden than FDG-PET/TC [197]. However, these differences were not consistently significant. As such, FDG-PET/CT should not be replaced but rather complemented by ^68^Ga-somatostatin receptor imaging when clinically indicated, aligning with the principles of personalized medicine [198]. Since neither SSTR- nor FDG-PET/CT consistently detects nodal MCC micro-metastases, these techniques are not intended to replace the sentinel lymph node biopsy.

### 7.5. Treatment of Metastatic Bone/Bone Marrow MCC

The treatment of metastatic bone/bone marrow Merkel cell carcinoma varies widely, ranging from single-modality approaches to multimodal regimens. Notably, except for a few studies that included patients treated with ICIs [7,128,132,135,136,138,141,143,147,156], the remaining published data relate to the pre-immunotherapy era (Table 4).

Among the 201 cases reviewed, chemotherapy (CHT) alone was utilized in 13 cases (6.4%) [129,131,137,143,144,148,155,159,164,165], with a median Merkel-specific survival (MSS) (IQR) of 8 (8) months. The treatment regimens mainly included platinum-based agents, often combined with etoposide [131,143,144,148,159,164]. Cyclophosphamide, typically in combination with doxorubicin or vincristine, was also utilized [137,148]. Additionally, other agents such as bleomycin [159], 5-fluorouracil, methotrexate [166,172], paclitaxel, and topotecan hydrochloride [131] were sporadically adopted. Like irinotecan (also known as CPT-11), topotecan, a topoisomerase I inhibitor, has been utilized to control tumor proliferation in refractory or palliative cancer settings, such as MCC or SCLC [199].

Furthermore, radiotherapy (RT) alone [133,165] and surgical intervention alone [152,158] were administered in two (1%) cases.

Multimodal approaches were the main adopted, with the use of CHT combined with RT described in 73 cases (36.3%) [6,130,138,145,147,151,154,157,161,162]. A comprehensive regimen including CHT, RT, and surgery was applied in 32 cases (16%) [126,160]., with immunotherapy utilized instead of CHT in 40 patients (20%) within the triple-therapy regimen group [7]. Dual-modality treatments were also documented, with CHT with immunotherapy in 11 cases (5.5%) [132,136], RT and surgery in 4 cases (2%) [134,149,150,153], and RT combined with immunotherapy in 2 cases (1%) [135,156].

Unfortunately, seven (3.4%) patients died before receiving the specific adjuvant treatment or immediately after surgical resection of the primary tumor [139,140,141,142,158,164,166]. For patients undergoing immunotherapy, the treatment involved ICIs, particularly Avelumab [135,136,156]. However, the specific agent used is not consistently reported [7,132].

The integration of immunotherapy into MCC treatment began with clinical trials in the early 2010s, focusing on ICIs targeting the PD-1/-L1 pathway. These studies demonstrated significant efficacy, leading to accelerated approvals by regulatory agencies (2017 for Avelumab, 2018 for Pembrolizumab, 2023/2024 for Retifanlimab), and transforming the treatment landscape for MCC with improved survival rates and durable responses compared to traditional chemotherapy. According to literature data, the objective response rate (ORR), the median progression-free survival (PFS), the OS, and the median OS results as follows: ~50–65%, ~8.3 months (Avelumab) and ~16.8 months (Pembrolizumab), 12- and 24-month OS rates of 60% and 37%, and ~20.3 months (Avelumab) for ICIs [200]; and 30–55% (first line), 3–4.6 moths, and 12- and 24-month OS rates of 47% and 20%, and ~9.5 months for CHT [201,202,203,204].

In metastatic MCC patients who discontinued ICI therapy for reasons other than disease progression, the risk of relapse varied significantly by response type: 24.9% in those achieving complete response (CR) versus 75.1% in non-CR patients [205,206].

Progression rates seemed also higher in MCPyV-positive MCC (70–75%) compared to MCPyV-negative cases (25–30%) [205]. This discrepancy may reflect differences in tumor immunogenicity and immune evasion mechanisms rather than inherent aggressiveness alone. MCPyV-negative tumors, despite having worse prognosis compared to positive alternatives, have a higher neoantigen load due to UV-induced mutations, potentially making them more responsive to ICIs. In contrast, MCPyV-positive tumors may have a lower tumor mutational burden and rely more on viral oncoproteins for oncogenesis, which might limit their immunotherapy responsiveness in some contexts, despite being less genomically unstable. Encouragingly, in cases of disease progression following ICI discontinuation, many patients, regardless of viral status, retained sensitivity to retreatment with the same immune checkpoint inhibitor [205].

In general, the management of metastatic bone MCC needs to be individually tailored. While surgery, potentially involving prosthetic replacement, may be an option for a single metastatic site, stereotactic hypofractionated RT should be considered for fit patients with oligometastatic (defined by a maximum of 3 to 5 metastatic sites) bone disease. Interestingly, a multivariate analysis by Gonzalez et al. revealed that MCC patients with bone metastases who underwent surgery to remove one of the metastatic sites (bone or other) had 0.92-times lower risk of death [122]. This finding aligns with the existing literature, which highlights that incorporating surgery into multimodal treatment can be a favorable prognostic factor for disease-free survival (DFS) in MCC [207,208].

Nevertheless, nowadays systemic immunotherapy is prioritized in the absence of contraindications with the available options, including Avelumab (anti-PD-L1 human monoclonal IgG1 antibody, FDA and EMA approved), Pembrolizumab (anti-PD-1 humanized monoclonal IgG4 antibody, FDA approved), Retifanlimab (anti-PD-1 humanized monoclonal IgG4 antibody, FDA and EMA approved), and Nivolumab (anti-PD-1 human monoclonal IgG4 kappa antibody, not FDA nor EMA approved). Given the limited published data, priority should be given to enrolling patients in clinical trials whenever possible, alongside decisions guided by a multidisciplinary treatment team following tumor board consultation.

## 8. MCC General Management

Treatment of Merkel cell carcinoma involves a combination of surgery, radiotherapy, and systemic immunotherapy, with this latter playing a significant role in improving patient outcomes and prognosis [209]. The standard of care for early-stage (stage I-II) MCC involves performing a wide local excision (WLE) or Mohs micrographic surgery (MMS), with MMS being preferred when WLE is impractical or for tumors located in the head and neck area to minimize the need for additional procedures [210,211,212,213,214]. After surgery, adjuvant radiotherapy (aRT) to the primary site is recommended for residual macroscopic (R2) (better if < 1 cm) or microscopic (R1) disease, or when mARFs are detected despite clear margins [2].

According to the current literature, aRT to the primary tumor bed is the standard of care and should be initiated as early as possible following surgery [215]. Consequently, in clinical practice, all patients regardless of the presence of high-risk features should be referred to a radiation oncologist to evaluate the indication for radiotherapy. To ensure timely initiation of treatment, margin-negative surgical techniques such as MMS should be preferred, when feasible, over more extensive, or reconstructive procedures that may delay the initiation of radiation [216].

A recent meta-analysis encompassing 17,179 MCC cases across 29 studies demonstrated a significant survival benefit associated with aRT. Patients receiving radiotherapy (78% stage I–II) showed improved OS (HR = 0.81; 95% CI: 0.75–0.86; *p* < 0.001) and DFS (HR = 0.45; 95% CI: 0.32–0.62; *p* < 0.001) compared to those who did not receive RT [217]. Subsequent studies seem to confirm these findings, demonstrating a reduced risk of loco-regional recurrence with the use of aRT in stage I-III MCC, regardless of patients’ immune status [218,219,220,221,222] and with 50 Gy in daily fractions of 2 Gy as a standard dose [223,224].

During surgery, a sentinel lymph node biopsy should be performed. If the results are negative, the decision between observation or aRT to the nodal basin should be made by a multidisciplinary team. Additionally, in cases where SLNB is unreliable—due to immunosuppression, anatomical constraints, or atypical lymph node drainage—or if SLNB is not feasible with risk of false negatives (e.g., patients with immunosuppression, unusual lymph node drainage, or multiple lymph node basins, such as in head and neck or midline trunk MCC) [2,3], aRT to both the primary site and the nodal basin should be considered.

However, the heterogeneity of the existing literature, along with the observed survival benefit of primary site aRT, but not nodal irradiation in stage III Merkel cell carcinoma, underscores the need for further research in this subset of patients [225].

Due to the high recurrence risk of stage III MCC, after SLNB or biopsy confirmation of clinically/imaging-detected nodal metastases, aRT to the nodal basin combined with complete lymph node dissection (CLND) is recommended, especially for patients with multiple affected lymph nodes or extranodal disease extension [3]. This approach aids in reducing recurrence and enhancing survival outcomes.

For patients with clinically evident nodal disease, the preferred treatment involves combined CLND plus RT, or clinical trials that incorporate neoadjuvant systemic therapy. Similarly, in cases of in-transit metastases, treatment typically includes surgery and/or RT, or participation in clinical trials [2]. For these patients, adjuvant CHT is not recommended [3].

Different approaches may be assessed for MCC of unknown primary (MCCUP), which typically is characterized by better outcomes than the primary known’ counterpart [118,226,227]. In these cases, after performing an FDG-PET/CT scan to rule out distant metastases, the management generally follows the same guidelines as for stage III known MCC [3]. However, due to the better prognosis associated with MCCUP, patients with exclusive nodal involvement may be candidates for CLND or RT alone, before considering a combined treatment approach.

Despite recent advancements in the diagnosis and treatment of MCC, locally advanced (stage III) and advanced (stage IV) disease can still be difficult to cure. For these cases, immunotherapy is recommended as a first- or second-line treatment [3].

In particular, Nivolumab may be proposed in the neoadjuvant setting when curative surgery or radiotherapy are not feasible, potentially allowing for surgical eligibility. Otherwise, systemic immunotherapy alone is also indicated with a preference for Avelumab and Pembrolizumab [2].

In case of recurrent locally advanced disease, Pembrolizumab and Retifanlimab should be indicated as treatment options over Avelumab [2].

**Figure 9:**
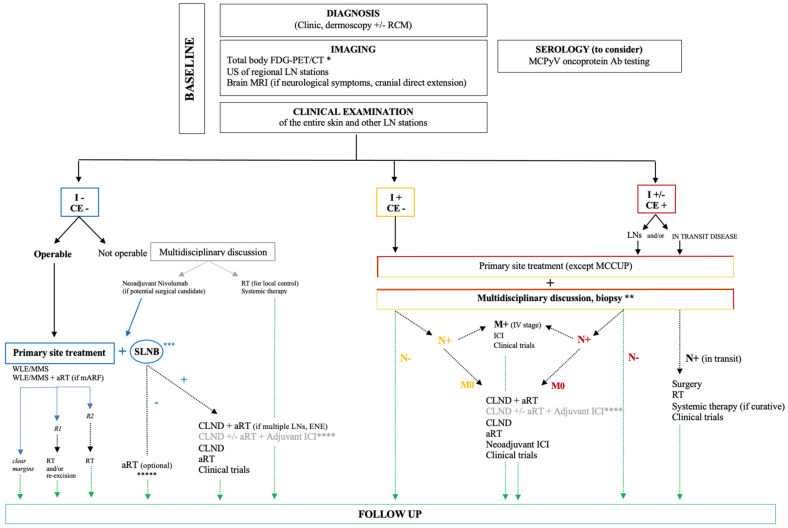
Evidence-based diagnostic and therapeutic flowchart for Merkel cell carcinoma. * In case of negativity, DOTA–peptide imaging may be considered. ** Confirmation biopsy: FNA, core needle, excisional biopsy; this last one may be considered to confirm a negative lymph node FNA or core needle biopsy. *** If SLNB is unreliable or not feasible, consider aRT to both the primary site and nodal basin. **** Future directions: ongoing clinical trials support the use of ICI in an adjuvant setting (NCT03271372, NCT04291885, NCT03712605). ***** False-negative SLNB outcomes may be in patients with immunosuppression, those with anatomical constraints, individuals with atypical LN drainage, in presence of multiple LNs basins (e.g., head and neck or midline trunk MCC). mARF resulting from the integration of current guidelines and evidence: tumor size ≥2 cm (or >1 cm per [3]), chronic immunosuppression (e.g., HIV, chronic lymphocytic leukemia, or solid organ transplant), head and neck primary sites, lymphovascular invasion, pathologically positive lymph nodes, or incomplete lymph node evaluation. Abbreviations used: Ab (Antibodies), aRT (adjuvant RadioTherapy), CE (Clinical Examination), CLND (Complete Lymph Node Dissection), ENE (ExtraNodal Extension), FNA (Fine Needle Aspiration), I (Imaging), ICI (Immune Checkpoint Inhibitor), LN/s (Lymph Node/s), mARF (modified Adverse Risk Factors), MCC (Merkel Cell Carcinoma), MCCUP (Merkel Cell Carcinoma of Unknown Primary), MCPyV (Merkel Cell PolyomaVirus), R1 (“microscopic residual disease”), R2 (“macroscopic residual disease”), RT (RadioTherapy), RCM (Reflectance Confocal Microscopy), SLNB (Sentinel Lymph Node Biopsy), WLE (Wide Local Excision).

Instead, in metastatic disease, all four mentioned ICIs, other than clinical trials, are viable treatment options [2,4]. Otherwise, CHT primarily consisting of platinum-based agents and etoposide is reserved for specific circumstances [2], while the addition of aRT to immunotherapy in histologically confirmed locally advanced/advanced-stage MCC (unresectable, recurrent, or metastatic) does not appear to enhance therapeutic efficacy [228]. A practical diagnostic–therapeutic flowchart following the current evidence is proposed in Figure 9.

Due to the aggressive nature of MCC and the limited systemic control of the disease, regular follow-up visits are crucial for patients’ outcome. Complete skin and lymph node examinations are recommended every 3–6 months for the first 3 years and every 12 months until the 5th year for primary tumors without additional high-risk factors. For patients with mARFs or those diagnosed from stage III onwards, a comprehensive and lifelong management plan should be sustained [4,124,209,210].

US of the primary scar, as well as the surrounding area and lymph nodes, should accompany clinical visits [4]. Additionally, stage III patients should also receive a whole-body FDG-PET/CT scan or contrast-enhanced CT scan of the neck, thorax, abdomen, and pelvis, along with brain MRI or CT (when indicated), every 3–6 months during the first 3 years. Afterward, follow-up imaging should take place every 6–12 months for the next 2 years.

For frail patients and those with stage IV disease, a personalized monitoring plan should be implemented [4].

Recent clinical trials have explored the use of adjuvant immunotherapy in surgically treated Merkel cell carcinoma to enhance systemic disease control. Adjuvant immunotherapy with Ipilimumab, when compared to observation in completely resected MCC, has been found to be ineffective in preventing disease progression and is associated with significant toxicity [229]. In contrast, Nivolumab has demonstrated a reduction in the absolute risk of recurrence, with DFS rates of 85% at 12 months and 84% at 24 months, compared to 77% and 73%, respectively, in the observation group [230].

Furthermore, a post hoc analysis of DFS by disease stage revealed that although median DFS was not reached for stage IIIA versus IIIB patients, at 48 months, stage IIIB patients receiving immunotherapy had a 70% two-year DFS, compared to just 32% in those who did not receive the adjuvant treatment [230].

Several ongoing clinical trials are investigating ICIs, either in combination with RT for advanced MCC or as monotherapy for earlier stages of the disease (NCT04291885, NCT03712605, NCT03271372). Notably, the phase 3 randomized, placebo-controlled ADAM trial (NCT03271372) is expected to provide crucial insights, evaluating Avelumab monotherapy as an adjuvant treatment for stage III MCC patients who have completed definitive therapy, including surgery and/or RT, for clinically detected metastases.

## 9. Conclusions

Merkel cell carcinoma (MCC) is a rare and aggressive neuroendocrine skin cancer, whose prognosis is still largely dependent on early diagnosis and accurate staging.

While two primary mechanisms (UV-induced and MCPyV-related) have been defined in MCC etiopathology and preclinical models suggesting a potential epithelial origin, comprehensive and definitive evidence is still lacking.

According to our results, the pattern of metastatic spread in MCC differs among studies, with bones being the third most common site of distant spread after the liver (second) and lymph nodes (first). Excluding head and neck MCC, which seems to be more regularly associated with liver metastases, the relationship between the primary tumor site and the development of bone or bone marrow metastases appears inconsistent. Furthermore, bone involvement does not reliably correlate with the poorest prognosis among metastatic sites. Nevertheless, the median OS for patients with metastatic bone/bone marrow MCC was 8 months (range 12.75–4), based on treatment strategies predominantly used before the introduction of immunotherapy.

Addressing the characteristics and impact of bone metastases (BMs), BMs exhibit a mixed biological (osteoblastic/osteolytic) and radiological behavior, with a marked preference for the axial skeleton over the appendicular skeleton. Pain and neurological symptoms are the most commonly observed, whereas leukemic spread during bone marrow disease in immunosuppressed patients may suggest a reasonable correlation.

Due to the absence of approved adjuvant treatments for systemic disease control following surgery/RT, early diagnosis of primary MCC through clinical assessment and non-invasive imaging techniques remains critical for improving patient outcomes. Moreover, routine baseline total-body imaging, including PET/CT scans and regional lymph node ultrasounds, is recommended to detect micro-metastatic or clinically occult disease, without replacing the SLNB procedure.

The integration of international guidelines, evolving evidence from clinical trials, and the expanding role of immune checkpoint inhibitors will contribute to improving systemic disease control and enhancing patient care.

## Figures and Tables

**Table 1:** Candidate cell of origin in Merkel cell carcinoma.

Cell of Origin	Driving Mechanisms	Evidence Supporting the Origin of MCC from the Specific Candidate Cell	
		Pros	Cons
**Merkel cell**	UV-related	Phenotypic similarities: CK 20, neuroendocrine markers (chromogranin A, SYA), Piezo2 and ATOH1.	No mitotic activity. No transformation/proliferation induced by MCPyV T antigens. Different anatomic localization between the candidate cell and MCC, with lack of connection between the tumor cells and epidermis.
**Epithelial** **progenitor**	UV-related	Presence of UV-signature (TP53, Rb inactivation). Ability to differentiate into Merkel cell and MCC. Shared mutations between in situ SCC and MCC in combined MCC/SCC tumors. Most likely origin of neuroendocrine carcinoma in other sites (SCLC).	Lack of connection between tumor cells and the epidermis.
**Epithelial** **follicular progenitor**	MCPyV- related	Similar DNA methylation profiles genes of UV-related MCC cell lines from epithelial origin. Common somatic mutations in combined trichoblastoma and MCPyV-MCC tumor. Expression of human MCC markers (dot-like KRT8 staining) and dermal localization of the tumors without connection to the epidermis/hair follicles in murine model.	Lack of UV signature. Genomic-level p53 inactivation in murine models, with no evidence of causation by MCPyV.
**Fibroblast**	MCPyV- related	Ability of MCPyV antigens to induce transformation in these cell types. Explain the exclusive dermal/hypodermal localization of MCC.	Lack of UV signature. No evidence of fibroblasts acquiring a Merkel cell-like phenotype. Unpredicted origin for neuroendocrine carcinoma.
**Pre/Pro B-cell**	MCPyV- related	Epidemiologic data on the association between MCC and B-cell neoplasia. Co-expression of B-cell markers (PAX5, TdT, Ig). Detection of MCPyV integration in B-cell neoplasia.	Lack of UV signature. No evidence of B-cells acquiring a Merkel cell-like phenotype. Unpredicted origin for neuroendocrine carcinoma.

**Table 2:** Immunohistochemical differential diagnoses of Merkel cell carcinoma.

Stain	MCC	SCLC	Other-Site SC Carcinoma	Neuroblastoma	Ewing Sarcoma	Small-Cell Melanoma	Lymphoma	Germ Cell Tumors
Neurofilament (NF)	+	−	−	+	+	−	−	−
Cytokeratin (CK) 20	+	−	Depending on the site	−	−	−	−	−
Cytokeratin (CK) 7	− *	+/−	Depending on the site	−	−	−	−	−
Thyroid transcription factor-1 (TTF-1)	+/−	+	−	−	−	−	−	−
Neuron-specific enolase (NSE)	+	+	+	+	+/−	−	−	−
Insulinoma-associated protein 1 (INSM1)	+	+	+	+/−	−	−	−	−
Chromogranin A	+	+	+	+	−	−	−	−
Synaptophysin (SYP)	+	+	+	+	+/−	−	−	−
Neural cell adhesion molecule (NCAM)/CD56	+	+	+	+	+/−	+	+/−	−
S100, SOC10, and other melanocytic markers	−	−	−	−	−	+	−	−
Leukocyte common antigen (LCA)/CD45 and other lymphocytic/lymphoblastic markers	−	−	−	−	−	−	+	−
Sal-like protein 4 (SALL4)	−	−	−	−	−	−	−	+
PRAME	+/−	−	−	−	+/−	+	−	+/−
SATB2	+	−	−	−	−	−	−	−
ALK	+/−	−	−	+/−	+/−	−	+/−	−
Rb	Loss in MCPyV-negative cases	Frequently loss	Frequently loss	Not loss	Not loss	Rarely loss	Not loss	Not loss
SOX2	+	−	−	+	+/−	−	−	It depends on the histotype
MCPyV large T-antigen (LTAg)	+ in MCPyV-positive cases	−	−	−	−	−	−	−

Positive (+): the marker is typically expressed in the tumor type; positive/negative (+/−): the expression may vary and is not definitive for that tumor type; negative (−): the marker is typically not expressed in the tumor type. * A small subset of CK7-positive, TTF-1-positive MCCs has been described.

**Table 3:** American Joint Committee on Cancer classification (AJCC) eighth edition for Merkel cell carcinoma.

AJCC Stage		TNM Staging	Primary Tumor	Lymph Node	Metastasis
0		Tis, N0, M0	In situ (within the epidermis only)	No regional lymph node metastasis	No distant metastasis
I	Clinical *	T1, N0, M0	≤2 cm maximum tumor dimension	Nodes negative by clinical exam (no pathological exam performed)	No distant metastasis
	Pathologic **	T1, pN0, M0	≤2 cm maximum tumor dimension	Nodes negative by pathologic exam	No distant metastasis
IIA	Clinical *	T2-3, N0, M0	>2 cm tumor dimension	Nodes negative by clinical exam (no pathological exam performed)	No distant metastasis
	Pathologic **	T2-3, pN0, M0	>2 cm tumor dimension	Nodes negative by pathological exam	No distant metastasis
IIB	Clinical *	T4, N0, M0	Primary tumor invades bone, muscle, fascia, or cartilage	Nodes negative by clinical exam (no pathological exam performed)	No distant metastasis
	Pathologic **	T4, pN0, M0	Primary tumor invades bone, muscle, fascia, or cartilage	Nodes negative by pathologic exam	No distant metastasis
III	Clinical *	T0-4, N1-3 *****, M0	Any size/depth tumor	Nodes positive by clinical exam (no pathological exam performed)	No distant metastasis
IIIA	Pathologic **	T1-4, pN1a(sn) *** or pN1a, M0	Any size/depth tumor	Nodes positive by pathological exam only (nodal disease not apparent on clinical exam)	No distant metastasis
		T0, pN1b, M0	Not detected (“unknown primary”)	Nodes positive by clinical exam, and confirmed via pathological exam	No distant metastasis
IIIB	Pathologic **	T1-4, pN1b-3, M0	Any size/depth tumor	Nodes positive by clinical exam, and confirmed via pathological exam OR in-transit metastasis ****	No distant metastasis
IV	Clinical *	T0-4, any N, M1	Any	+/− Regional nodal involvement	Distant metastasis detected via clinical exam
	Pathologic **	T0-4, any pN, M1	Any	+/− Regional nodal involvement	Distant metastasis confirmed via pathological exam

* Clinical detection of nodal or metastatic disease may be via inspection, palpation, and/or imaging. ** Pathological detection/confirmation of nodal disease may be via sentinel lymph node biopsy, lymphadenectomy, or fine needle biopsy; and pathological confirmation of metastatic disease may be via biopsy of the suspected metastasis. *** (sn) = sentinel lymph node. The N1 category defines a regional lymph node metastasis without in-transit metastasis. The N1 category is subdivided into N1a(sn) for clinically occult lymph node metastasis detected only at SLNB in a patient with clinical stage I-II disease; N1a for clinically occult lymph node metastasis detected following lymph node dissection; and N1b for clinically and/or radiologically detected metastasis. **** In transit metastasis: a tumor distinct from the primary lesion and located either (a) between the primary lesion and the draining regional lymph nodes or (b) distal to the primary lesion. ***** N2 is defined as in-transit metastasis without associated lymph node metastasis; N3 is defined as in-transit metastasis with lymph node metastasis.

**Table 4:** Summary of the studies investigating bone and bone marrow metastases from Merkel cell carcinoma: patient characteristics, nature of the primary MCC, treatment, pattern of metastatic spread, and overall survival.

Author	Study’ Type	Patient/s				Merkel Cell Carcinoma				Other Site/s of Distant Metastasis (n., %) *	OS for Bone/BM Metastatic Patients
		Number (n.)	Age (Years)	Sex		Primary MCC		Bone/BM Metastases			
						Known	Unknown				
				Male n (%)	Female n (%)	n. (%)	n. (%)	n. (%)	Therapy		
Lewis et al. [6]	Original article	215	/	176 (82)	39 (18)	173 (80)	42 (20)	64 (21)	CHT, RT	Non-regional LNs (88, 41%)	/
Kim et al. [7] *****	Original article	151	76 (62) ***	101 (66.9)	50 (23.1)	134 (88.8)	17 (11.2)	40 (26.5)	IT, RT, surgery	LNs (94, 62.3), skin/soft tissue (40, 26.5)	15.1 months (median)
Maloney et al. [125]	Original article	331	74.6 (15.5) ***	241 (72.8)	90 (27.2)	****	****	6 (1.9)	/	Liver (89, 28.7), lung (51, 16.4), brain (6, 1.9)	5-year median OS rate of 11.2%
Xia et al. [126]	Original article	273	****	200 (73.3)	73 (26.7)	184 (67.4)	89 (32.6)	31 (11.3) **	CHT, RT, surgery	Liver (37, 13.5)	1-year median OS rate of 38.7% **
Wang et al. [127]	Case report #	1	79	/	1 (100)	/	1	1 BM	/	/	/
Keow et al. [128] *****	Case report #	1	71	1 (100)	/	1	/	1 BM	/	/	/
Goepfert et al. [129]	Original article	41	66 (55) ***	****	****	****	****	4 (9.8)	CHT	Skin (5, 12.1%), LNs (4, 9.8%)	/
Haykal et al. [130]	Case report #	1	49	/	1 (100)	1 Vulva	/	1 Intradural *intramedullary* C4-C5	CHT, RT	Regional and non-regional LNs, liver	-
Barkdull et al. [131]	Case report #	1	55	1 (100)	/	1 Scalp	/	1 Sternum	CHT	Regional LNs, subcutaneous tissue, pancreas	9 months
Khaddour et al. [132] *****	Original article	34	70.2 (51.4) ***	20 (58.8)	14 (41.2)	14 (41.2)	20 (58.8)	10 (29.4)	CHT, IT	Regional LNs (28, 82.4)	8.2 months (median)
Payne et al. [133]	Case report #	1	77	/	1 (100)	1 Buttock	/	1 T4 vertebra	RT	Bone, lung	12 months
Abul-Kasim et al. [134]	Case report #	1	65	1 (100)	/	/	1	1 Epidural and intradural L1, L5	RT, surgery	Non-regional LNs, brain, retroperitoneum, lung	8 months
Pennisi et al. [135] *****	Case report #	1	73	/	1 (100)	1 Face	/	1 Intradural *extramedullary* C6-C7	IT (Avelumab), RT	Skin, subcutaneous tissue	5 months
Leão et al. [136] *****	Case report #	1	61	1 (100)	/	1 Buttock	/	1 Sacrum	CHT, IT (Avelumab)	In-transit metastasis	30 months
Lentz et al. [137]	Case report #	1	55	1 (100)	/	1 Scalp	/	1 BM	CHT	Regional LNs, parotid gland	12 months
Khan et al. [138] *****	Case report #	1	80	/	1 (100)	1 Trunk	/	1 BM	CHT, RT	Regional LNs	1 month
Morris et al. [139]	Case report #	1	72	1 (100)	/	1 Shoulder	/	1 BM	Death before starting CHT	Regional LNs	4 months
Kressin et al. [140]	Case report #	1	64	1 (100)	/	1 Forehead	/	1 BM	Death before starting CHT	Regional LNs	3 months
Durmus et al. [141] *****	Case report #	1	60	1 (100)	/	1 Thigh	/	1 BM	Death before starting IT	Regional LNs, liver	7 months
Nemoto et al. [142]	Case report #	1	73	/	1 (100)	1 Cheek	/	1 BM	Death before starting therapy	Regional LNs	8 months
Highland et al. [143] *****	Case report #	1	74	1 (100)	/	1 Lip	/	1 BM	CHT	Regional LNs	13 months
Smadja et al. [144]	Case report #	1	34	/	1 (100)	1 Shoulder	/	1 BM	CHT	Lung, brain	4 months
Le Gall-Ianotto et al. [145]	Case report #	1	65	1 (100)	/	/	1	1 BM	CHT, RT	/	3 months
Kobrinski et al. [146]	Case report #	1	86	1 (100)	/	1 Trunk	/	1 BM	RT	Regional LNs	12 months
Folyovich et al. [147] *****	Case report #	1	62	/	1 (100)	1 Arm	/	1 skull	CHT, RT	Non-regional LNs	24 months
Vlad et al. [148]	Case report #	1	72	1 (100)	/	1 Arm	/	1 BM	CHT	Regional LNs	8 months
Goodwin et al. [149]	Case report #	1	76	1 (100)	/	1 Back	/	1 Epidural T5	RT, surgery	Bone	15 months
Madden et al. [150]	Case report #	1	55	1 (100)	/	1 Neck	/	1 Epidural T6-T8	RT, surgery	Bone	4 months
Moayed et al. [151]	Case report #	1	70	1 (100)	/	/	1	1 Lumbosacral spine, epidural S1, hip	CHT, RT	Regional LNs	9 moths
Nguyen et al. [152]	Case report #	1	69	1 (100)	/	1 Cheek	/	1 Tibia	Surgery	/	19 months
Kamijo et al. [153]	Case report #	1	75	/	1 (100)	1 Cheek	/	1 Femur	RT, surgery	Subcutaneous tissue	16 months
Pectasides et al. [154]	Case report #	1	48	1 (100)	/	1 Buttock	/	1 T11, L2 vertebra	CHT, RT	Regional LNs	5 months
Pilotti et al. [155]	Original article	50	62 (45) ***	22 (44)	28 (56)	40 (80)	10 (20)	1 (2)	CHT	Skin (4, 8), liver (2, 4), pancreas (2, 4), lung (1, 2)	12 months
Principe et al. [156] *****	Case report #	1	79	1 (100)	/	1 Ear	/	1 T2, T7, T10-11, L3 vertebra	IT (Avelumab), RT	Regional LNs, parotid gland	18 months
Vijay et al. [157]	Case report #	1	57	/	1 (100)	/	1	1 Extra-dural T8, L4, S1	CHT, RT	Non-regional LNs	1 month
Ng et al. [158]	Case report #	1	73	1 (100)	/	1 Arm	/	1 Extra-dural T5-T7	Surgery, death before starting CHT/RT	/	1 month
Turgut et al. [159]	Case report #	1	63	1 (100)	/	1 Abdomen	/	1 Extradural L5–S1	CHT	“Massive” ****	2 months
Zhao et al. [160]	Case report #	1	54	1 (100)	/	/	1	1 T6, T12, L2 vertebra	CHT, RT, surgery	Regional LNs, liver	21 moths
Maugeri et al. [161]	Case report #	1	59	/	1(100)	1 Scalp	/	1 T7-T8 vertebra	CHT, RT	Liver, lung	8 months
Chao et al. [162]	Case report #	1	23	/	1 (100)	1 Back	/	1 Extradural T3-T4	CHT, RT	Lung, heart	23 months
Turgut et al. [163]	-	-	-	-	-	-	-	-	-	-	-
Tam et al. [164]	Case report #	1	66	1 (100)	/	1 Forearm	/	1 BM	Death before therapy	/	6 months
-	-	1	55	1 (100)	/	/	1	1 BM	CHT	/	1.5 month
Gooptu et al. [165]	Case report #	1	68	/	1 (100)	1 Leg	/	1 BM	CHT	Non-regional LNs	2 months
-	-	1	55	1 (100)	/	1 Neck	/	1 Vertebra	RT	Non-regional LNs, brain	6 months
Park et al. [166]	Case report #	1	30	1 (100)	/	1 Hand	/	1 C6 vertebra	Death before starting CH	/	1 month

Legenda: BM (bone marrow), CHT (chemotherapy), IT (immunotherapy), LNs (lymph nodes), MCC (Merkel cell carcinoma), OS (overall survival), RT (radiotherapy). **#** For individual case reports, we specified the anatomical site involved in the “Bone metastasis” section when provided; age is recorded in years, and prognosis in months. * We included the most reported metastatic site/s alongside bone. ** We considered only single bone-site metastases. *** Median age (IQR). **** Unable to determine due to limitations in the available published data. ***** Studies including patient/s diagnosed and treated after the advent of immunotherapy. / None or not intended by the study.

## Data Availability

No new data were created or analyzed in this study. Data sharing is not applicable to this article.

## Abbreviations

The following abbreviations, not otherwise defined in the main text, are used in this review:

AIDS	Acquired Immunodeficiency Syndrome
ALK	Anaplastic Lymphoma Kinase
AKT1	v-akt murine thymoma viral oncogene homolog 1
ARID1	AT-Rich Interactive Domain-Containing Protein 1
ATM	Ataxia-Telangiectasia Mutated
ASXL1	Additional Sex Combs-Like 1
BCOR	BCL6 Corepressor
BRCA1/2	Breast Cancer 1/2
CDX2	Caudal-type Homeobox 2
DNA	Deoxyribonucleic Acid
DOTA	1,4,7,10-Tetraazacyclododecane-1,4,7,10-tetraacetic acid
EMA	European Medicines Agency
EP400 complex	E1A-binding protein p400 complex
EZH2	Enhancer of Zeste Homolog 2
FAT1	FAT Atypical Cadherin 1
FDA	Food and Drug Administration
GATA3	GATA Binding Protein 3
Gy	Gray
HRAS	Harvey Rat Sarcoma Viral Oncogene Homolog
HIV	Human Immunodeficiency Virus
HMB45	Human Melanoma Black 45
HR	Hazard Ratio
ICI	Immune Checkpoint Inhibitor
IQR	Interquartile Range
ISL1	ISL LIM Homeobox 1
JAK-STAT	Janus Kinase-Signal Transducer and Activator of Transcription
KMT2	Lysine Methyltransferase 2
KRT	Keratin
LSD1	Lysine-Specific Demethylase 1
MAPK	Mitogen-Activated Protein Kinase
MDM2	Mouse Double Minute 2 homolog
MSH2	MutS Homolog 2
MYCL	v-myc avian myelocytomatosis viral oncogene lung carcinoma-derived homolog
NCCN	National Comprehensive Cancer Network
NKX3.1	NK3 Homeobox 1
NOTCH1	Notch homolog 1
PAX5	Paired Box 5
Piezo2	Piezo-type mechanosensitive ion channel component 2
PD-1/-L1	Programmed Death-1/Programmed Death-Ligand 1
PIK3CA	Phosphatidylinositol-4,5-Bisphosphate 3-Kinase Catalytic Subunit Alpha
PI3K	Phosphoinositide 3-Kinase
POU3F2	POU Class 3 Homeobox 2/BRN2
PRAME	Preferentially Expressed Antigen in Melanoma
SATB2	Special AT-rich Sequence-Binding Protein 2
SD	Standard Deviation
SMARCA4	SWI/SNF Related, Matrix Associated, Actin Dependent Regulator of Chromatin Subfamily A, Member 4
SOX	SRY-related HMG-box family of transcription factors
STIR	Short Tau Inversion Recovery
SUV	Standardized Uptake Value
TdT	Terminal deoxynucleotidyl Transferase
VP1	Viral Protein 1
